# Maternal stress in the Neonatal Intensive Care Unit: A concept analysis

**DOI:** 10.1111/jjns.70039

**Published:** 2026-01-01

**Authors:** Mitsuki Nojima, Hisayo Okayama

**Affiliations:** ^1^ Doctor Program in Nursing Science, Graduate School of Comprehensive Human Sciences University of Tsukuba Ibaraki Japan; ^2^ Institute of Medicine University of Tsukuba Ibaraki Japan

**Keywords:** concept analysis, mother, NICU, stress

## Abstract

**Aims:**

This study aimed to provide a clear definition of the concept of maternal stress in the neonatal intensive care unit (NICU), which has been inconsistently described in previous studies. It also aimed to identify the essential attributes, antecedents, and consequences of maternal stress through concept analysis.

**Methods:**

Rodgers' concept analysis method was used. A search was conducted using the web version of the Central Journal of Medicine, PubMed, PsycINFO, and CINAHL, yielding 223 hits. Of these, 32 were selected for the analysis.

**Results:**

Three attributes of maternal stress in the NICU were identified: “Earnest feelings for my infant,” “Stuck feeling,” and “Vague anxiety about the future.” Six antecedents and four consequences were identified.

**Conclusions:**

The results of this study indicated that mothers felt a sincere desire for their infants, as well as a vague sense of helplessness due to circumstances beyond their control, and anxiety about the future. These findings suggest that while the NICU environment can be a source of stress for mothers, it may also serve as a supportive space and an opportunity for them to reflect on their role as mothers.

## 1 INTRODUCTION

According to the findings of the United Nations International Children's Emergency Fund (UNICEF) and the World Health Organization (WHO), approximately 15% of newborns worldwide have low birth weight (United Nations Children's Fund (UNICEF) & World Health Organization, 2019). As these infants require immediate treatment and care in the neonatal intensive care unit (NICU), they are separated from their mothers immediately after birth. This separation can lead to increased anxiety, depression, and stress among mothers (Shetty et al., 2024). Fathers may also experience similar emotions; however, research has demonstrated that mothers frequently exhibit elevated levels of anxiety, depression, and stress in comparison to fathers (Caporali et al., 2020; Shetty et al., 2024). Moreover, approximately one‐third of mothers of infants admitted to the NICU have been observed to manifest post‐traumatic stress disorder (PTSD) because of their NICU experience (McKeown et al., 2023). In this regard, research indicates an elevated incidence of PTSD in mothers compared to fathers (Aftyka et al., 2017). Anxiety and stress symptoms have been demonstrated to negatively impact mother‐infant bonding; depressive symptoms during NICU admission are predictors of depressive symptoms at two to three months postpartum (Bonacquisti et al., 2020). This suggests a potential concern: insufficient maternal care during the NICU episode may have negative consequences not only for the mother but also for the infants. Consequently, healthcare professionals in the NICU play a pivotal role not only in preserving the life of the infants but also in providing comprehensive care for the attending family, particularly the mother. Nevertheless, NICU settings face many systemic and environmental challenges. The NICU environment is characterized by the constant presence of medical devices and alarms, which can be distressing for mothers visiting their infants. Some studies have reported that the lack of private space can be emotionally taxing for mothers, as it may lead to unintended exposure to the distress or conversations of other families within the shared environment (Bry & Wigert, 2019). Another study found that one of the challenges in NICUs is the inadequate respect for the privacy of mothers and infants (Maleki et al., 2024). Previous studies have indicated that both the physical and organizational characteristics of NICUs and family stress and anxiety can hinder the implementation of family‐centered care (FCC; Abukari & Schmollgruber, 2024; Aljawad et al., 2025). In particular, high levels of stress and anxiety among family members can reduce their participation in caregiving and communication with healthcare staff, thereby limiting the practice of FCC. Because FCC benefits both mothers and infants (De Bernardo et al., 2017), healthcare providers need to address both environmental and family‐related factors to promote FCC.

Maternal psychological burden is commonly expressed with words such as anxiety, fear, and stress. Of these, stress is defined as “the physiological or psychological response to internal or external stressors” (American Psychological Association, n.d.). External triggers, known as stressors, elicit stress responses in individuals, which are mediated by cognitive appraisal and expressed through coping mechanisms (Lazarus & Folkman, 1984). Based on this theory, most previous studies of mothers of infants admitted to the NICU focused on specific stress reactions such as anxiety and fear (Grieb et al., 2023; Suzuki et al., 2016). However, these studies only viewed stress as an emotional “outcome” and did not fully consider the process as a whole, including the stressor and evaluation process.

Furthermore, even when faced with the same stressor, mothers may differ in their perceptions and responses. Therefore, focusing solely on individual stress responses, such as anxiety or fear, is insufficient to fully capture the complexity of maternal stress experiences. Accordingly, a more comprehensive understanding of maternal stress would require consideration of the entire process, from stressors to stress responses.

Therefore, this study aimed to provide a clear definition of the concept of maternal stress in the NICU, which has been inconsistently described in previous studies (Grieb et al., 2023; Suzuki et al., 2016). In addition, the study sought to clarify the essential attributes, antecedents, and consequences of this concept through concept analysis. While systematic or scoping reviews synthesize prior findings, they do not provide a theoretical definition of a concept. Concept analysis, in contrast, is specifically intended to define concepts based on their essential attributes (Rodgers & Knafl, 2000). Because maternal stress in the NICU had not been theoretically defined, concept analysis was considered the most appropriate method for this study. By clarifying the factors that mothers respond to and how they react psychologically, it is possible to identify directions for more individualized support. Furthermore, a deeper understanding of maternal stress is expected to contribute to the promotion of FCC. Therefore, this study aimed to clarify the concept of maternal stress in the NICU through concept analysis to provide a theoretical definition and identify its essential attributes, antecedents, and consequences.

## 2 METHODS

### 2.1 Study design

This study used Rodgers' method of conceptual analysis (Rodgers & Knafl, 2000). Walker and Avant's (2005) method is also well known for conceptual analysis. The latter method aims to distinguish similarities and differences between concepts by examining their underlying elements and revealing their internal structure, which is also useful for creating measurement tools and interview guides (Walker & Avant, 2005). In contrast, Rodgers' method views concepts as constantly changing objects, and is described as “a heuristic method for clarifying and further exploring the current use of concepts with attention to contextual and temporal aspects” (Rodgers & Knafl, 2000). The purpose of this study was to explore the components and characteristics of maternal stress, rather than to develop a measurement instrument or interview guide. Moreover, the stress experienced by mothers of infants admitted to the NICU occurs in the contextual and temporally variable environment of the NICU. Therefore, Rodgers' conceptual analysis was chosen as the analytical method that would best serve the purpose of this study.

### 2.2 Literature collection methods

The term “stress” is employed not only in the domain of nursing science but also in various other fields, including psychology, behavioral science, and social science. Four databases were used to collect literature in these fields: the Central Journal of Medicine web, PubMed, PsycINFO, and CINAHL. The search formula was designed to encompass relevant keywords, such as “NICU,” “mother,” and “stress,” and was formulated as follows: (“neonatal intensive care unit” or “NICU”) and “mother” and “stress.” The inclusion criteria were (1) articles written in English or Japanese and (2) articles published in or after the year 2000. The exclusion criteria were (1) articles that did not address maternal stress in the NICU and (2) articles focusing on the effects of interventions such as programs or educational media. Although gray literature is generally included in systematic reviews, it was excluded from this study because of its low search reproducibility, leading to a lack of objectivity in literature selection, and the difficulty of ensuring research quality when non‐peer‐reviewed literature is included. The concept analysis was limited to peer‐reviewed academic papers because the purpose of this study was to clarify the definition of the concepts of maternal stress in the NICU. We also searched for publication types without any restrictions. The first author searched the database in September 2024.

**FIGURE 1:**
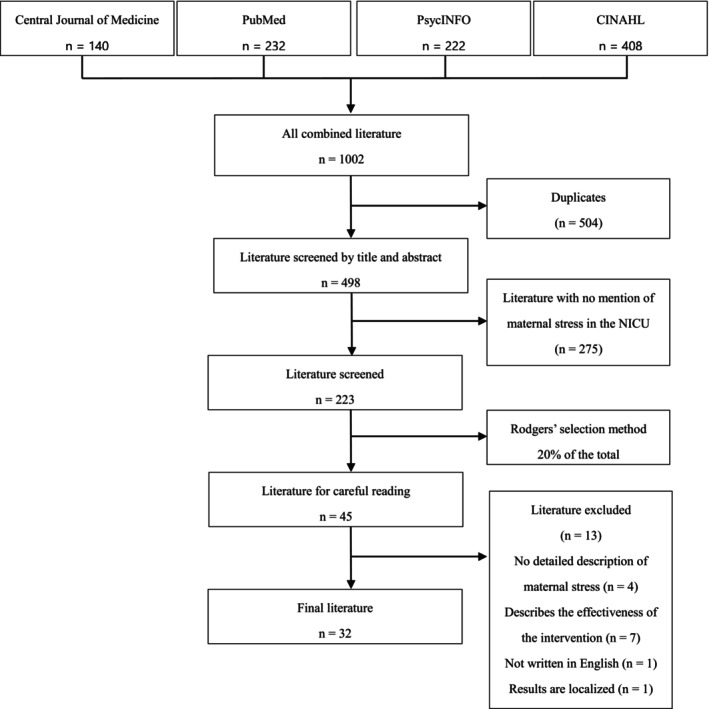
Literature selection process.

The selection process is shown in Figure 1. The first author selected the studies based on the inclusion and exclusion criteria. First, duplicates were excluded from all literature hits in the four databases. Second, the titles and abstracts of the studies were read, and those that did not mention maternal stress in the NICU were eliminated. Ten articles were found on the Central Journal of Medicine Web, and 213 were found in PubMed, PsycINFO, and CINAHL, for a total of 223. According to Rodgers and Knafl (2000), “a minimum of 30 items from each discipline or hierarchy or 20% of the population, whichever is greater, should be selected.” Using this as a guideline, a random selection of 45 papers, or 20% of the 223 hits, was obtained using a computer program. Finally, the papers were selected through a comprehensive review of the entire text of the 45 cases. Although the title and abstract mentioned maternal stress, 13 papers met the exclusion criteria based on the full‐text reading. After excluding these 13 papers, a total of 32 articles were included in the analysis. In accordance with Rodgers and Knafl (2000), no additional literature search was conducted because the minimum number of references required for concept analysis was 30. All references were imported and managed using Refworks. In cases for which the selection decision was uncertain, consultation was sought with the second author to ensure the reliability of the literature.

### 2.3 Analysis

Based on Rodgers and Knafl (2000) method of conceptual analysis, we sought to clarify maternal stress in the NICU in terms of its antecedents, attributes, and consequences. Specifically, the following five steps were performed.

1. Articles describing maternal stress in the NICU were identified. We then extracted the descriptions of maternal stress from the articles. During the extraction, several studies included in the analysis addressed both mothers and fathers. Therefore, only those statements that explicitly referred to mothers' experiences were extracted. For cases in which it was unclear whether a statement referred specifically to mothers, the matter was discussed with the second author until consensus was reached
2. The extracted data were organized into a matrix table. Factors reported in the literature as causing stress were identified as antecedents, stress reactions directly arising from these factors were recognized as attributes, and subsequent reactions were interpreted as consequences.
3. Each dataset was inductively grouped into extracts with similar semantic content and labeled.
4. Related concepts and surrogate terms for this concept were identified
5. Maternal stress in the NICU was defined based on prior antecedents, attributes, and consequences.

To ensure the integrity of the analysis, the sections of the paper and the analysis results were repeatedly reviewed. Throughout the analysis process, the results were discussed with the second author, who is versed in conceptual analysis, to ensure the validity of the findings.

## 3 RESULTS

### 3.1 Overview

**FIGURE 2:**
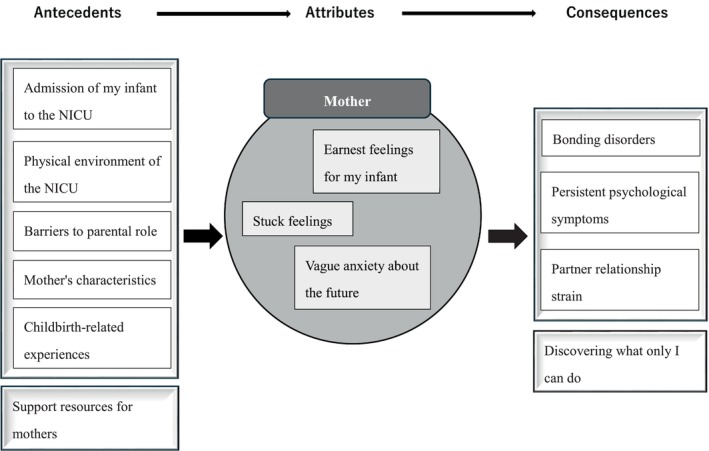
Conceptual model of maternal stress in the neonatal intensive care unit (NICU) (antecedents, attributes, and consequences).

The characteristics of the 32 references obtained from our search are shown in Table 1. Of these, 13 were published after 2019, indicating a recent increase in research focusing on maternal stress in the NICU. Upon review of the 32 references, 40 subcategories and 76 codes were extracted. Consequently, the analysis yielded six antecedents, three attributes, and four consequences, as illustrated in Figure 2.

**TABLE 1:** Literature list.

No.	Author	Country	Research design	Population	NICU level	Findings
1	Aftyka et al. (2017)	Poland	Observational study	125 parents (72 mothers and 53 fathers)	NA (third‐referral neonatal intensive care unit)	Compared to the fathers, the mothers felt greater stress and presented a higher severity of post‐traumatic stress disorder.
2	Aftyka et al. (2014)	Poland	Cross‐sectional observational study	39 mothers and 27 fathers	NA (children's university hospital)	Mothers reported significantly higher severity of PTSD symptoms, particularly intrusion and arousal symptoms.
3	Al‐Akour et al. (2015)	Jordan	Cross‐sectional study	150 mothers (75 NICU group, 75 non‐NICU group)	NA (three large hospital)	The results showed that mechanical ventilation, lower birth weight, and lower gestation age were predictors of higher anxiety level and depression among mothers of infants admitted to the NICU.
4	Bonacquisti et al. (2020)	United States	Prospective longitudinal observational study	127 mothers	NA (three hospitals)	Maternal–infant attachment was negatively associated with anxiety and stress symptoms.
5	Caporali et al. (2020)	NA (Meta‐analysis including data from 31 countries)	Meta‐analytic study	14,875 parents (146 studies)	NA (varies by included study)	Mothers report higher NICU‐related stress than fathers.
6	D'Souza et al. (2009)	India	Descriptive cross‐sectional survey	62 mothers, 38 fathers	Tertiary level NICU	Nursing support was mildly negatively correlated with parental stress.
7	Fernández Medina et al. (2019)	Spain	Qualitative, interpretative design	15 mothers	Level‐III	The subcategory “My job was to make milk” was extracted, which indicated that mothers felt an obligation or stress from providing breast milk.
8	Fróes et al. (2020)	Brazil	Cross‐sectional study	74 mothers	Level‐III	“Alteration in parental roles” was identified as the main source of stress experienced by mothers.
9	Greene et al. (2015)	United States	Prospective study	69 mothers	Level‐III	The associations between distress and maternal visitation are substantial, with apparent lasting associations between visitation and long‐term maternal distress.
10	Grieb et al. (2023)	United States	Qualitative Study	26 mothers	NA (two urban hospital NICUs)	Most of the mothers described their experience in the NICU as overwhelming and traumatizing, filled with stress, uncertainty, sadness, guilt, and isolation.
11	Guillaume et al. (2013)	France	Qualitative study	30 mothers, 30 fathers	NA (three tertiary care centers)	The nurses' caring attitude toward the baby and parents, and their communication with parents, reduced stress and made interactions with the baby possible.
12	Hatters Friedman et al. (2013)	United States	Descriptive observational study	150 mothers	NA (babies and children's hospital)	Mothers were more likely to receive psychiatric treatment in the NICU if they had longer infant hospitalizations, were younger in age, had fewer living children, and had lower levels of education.
13	Holditch‐Davis and Miles (2000)	United States	Qualitative study	31 mothers	Tertiary university‐based NICU	The study indicates that health care providers, and especially nurses, can have a major role in reducing parental distress by maintaining ongoing communication with parents and providing competent care for their infants.
14	Holditch‐Davis et al. (2003)	United States	Qualitative study	30 mothers	Tertiary university‐based NICU	All mothers interviewed had at least one posttraumatic symptom, 12 had two, and 16 had three symptoms.
15	Holditch‐Davis et al. (2015)	United States	Longitudinal study	232 mothers of preterm infants	NA (four hospitals)	The mother experiences persistent stress due to the baby's admission to the NICU.
16	Kaur et al. (2022)	India	Descriptive study	17 parents	NA (tertiary care teaching hospital)	Mothers and fathers were very stressed during the period of hospitalization of their preterm neonates.
17	Kim et al. (2020)	South Korea	Cross‐sectional study	294 mothers	Tertiary hospitals	Maternal self‐representation was the strongest predictor of maternal postpartum attachment.
18	Lee et al. (2005)	United States	A cross‐sectional, descriptive, correlational study	30 Chinese‐American families (30 mothers, 25 fathers)	NA (three ICU sites)	Lack of support from healthcare providers significantly contributed to parental stress.
19	Levinson et al. (2022)	United States	Prospective study	135 mothers	NA (regional perinatal center)	Maternal stress, measured by PSS: NICU, was significantly associated with PPD screening outcomes.
20	Lotterman et al. (2019)	Columbia	Longitudinal study	91 mothers	Level‐IV	This study noted elevated rates of psychological symptoms in mothers of moderate‐ to late‐preterm infants over time.
21	Suzuki et al. (2016)	Japan	Longitudinal study	100 mothers	NA (university hospital)	There is a negative correlation between social support and the mother's mental and physical stress responses.
22	Ong et al. (2019)	Malaysia	Cross‐sectional descriptive study	180 mothers	NA (public hospital)	The infant's NICU admission will trigger stress in mothers.
23	Orovou et al. (2023)	Greece	Prospective observational study	469 postpartum women	NA (university hospital)	NICU admission significantly increases the risk of postpartum PTSD.
24	Reimer et al. (2023)	Sweden	Mixed‐methods study	437 mothers and 301 fathers	Level‐III	Higher NICU‐related stress was significantly associated with increased relationship strain.
25	Rossman et al. (2013)	United States	Qualitative descriptive design	23 mothers	Level‐III	Mothers had faith in the healing properties of their milk and equated providing milk with “giving life” to their infants.
26	Schappin et al. (2013)	United States	Meta‐analytic study	3025 parents (38 studies)	NA (varies by included study)	The results indicate that parents of preterm‐born children experience only slightly more stress than parents of term‐born children.
27	Schecter et al. (2020)	United States	Cross‐sectional survey study	80 parents	NA	15% of parents reported moderate to high severity PTSD symptoms, more than 1 year after the NICU experience.
28	Shaw et al. (2009)	United States	Prospective longitudinal study	18 parents (11 mothers and 7 fathers)	NA (children's hospital)	At the 4‐month follow‐up, 18% of parents had high levels of PTSS.
29	Siva et al. (2024)	India	Meta‐analytic study	descriptive quantitative studies (*n* = 1889) and qualitative study (*n* = 173)	NA (varies by included study)	The systematic review and meta‐analysis findings show that the neonatal condition, parental role alteration, NICU environment, and communication gaps were stressors for mothers of neonates admitted to the NICU in India.
30	Stotts et al. (2019)	United States	RCT (longitudinal mediation analysis)	360 postpartum mothers	NA (urban children's hospital)	Psychological inflexibility measured 2 weeks after infant discharge from the hospital fully mediated the relationship between early and later depressive symptoms at 3 months postpartum.
31	Nakazawa et al. (2006)	Japan	Descriptive observational study	20 mothers	NA (seven hospitals)	Mothers' stress increases when the NICU is the place where they meet their baby for the first time.
32	Woodward et al. (2014)	New Zealand	Longitudinal cohort study	133 mothers	Level‐III	Parental role alteration is the most stressful NICU‐related factor for mothers. Higher NICU‐related maternal stress was linked to poorer child outcomes at age 4 years.

Abbreviations: ICU, intensive care unit; NA, not available; NICU, neonatal intensive care unit; PPD, postpartum depression; PTSD, post‐traumatic stress disorder; PTSS, post‐traumatic stress symptoms; RCT, randomized controlled trial.

### 3.2 Antecedents

The six antecedent categories identified are displayed in Table 2. The “Admission of my infant to the NICU” was found to be separated by their own infant's NICU admission (Holditch‐Davis & Miles, 2000; Ong et al., 2019; Schecter et al., 2020), and by prolonged hospitalization (Caporali et al., 2020; Hatters Friedman et al., 2013; Siva et al., 2024), which can trigger maternal stress reactions. Furthermore, it was observed that the infant's presence in the NICU itself was a stressor for the mother (Al‐Akour et al., 2015; Caporali et al., 2020; Kaur et al., 2022). The “Physical environment of the NICU” encompassed the NICU environment itself, which was characterized by the presence of medical equipment and alarms (Fernández Medina et al., 2019; Kim et al., 2020). For example, Grieb et al. (2023) reported that the NICU environment, filled with machines and alarm sounds, heightened maternal stress. One mother described her experience by saying, “*I feel like I'm this cat ready to pounce because I'm just staring at the monitors*,” illustrating the constant vigilance and tension induced by the surrounding medical equipment. The “Barriers to parental roles” were expressed as stressors, that is, situations that limited mothers' actions. Specifically, these barriers manifested as the inability to nurture their infants according to their initial expectations (Grieb et al., 2023; Holditch‐Davis & Miles, 2000; Siva et al., 2024), the incapacity to relieve their infants' pain (Fróes et al., 2020; Siva et al., 2024), and the inability to interact with their infants (Guillaume et al., 2013). The literature indicates that learning new healthcare systems and symptoms, as well as abrupt changes in lifestyle and plans, are also stressors for mothers (Grieb et al., 2023). “Mother's characteristics,” including being younger (Hatters Friedman et al., 2013; Schappin et al., 2013), less educated (Hatters Friedman et al., 2013; Holditch‐Davis & Miles, 2000; Woodward et al., 2014), having psychological distress (Holditch‐Davis et al., 2003; Woodward et al., 2014), and having economic constraints (Siva et al., 2024), were found to intensify mothers' stress responses. In the “Childbirth‐related experiences” category, having a previous miscarriage (Holditch‐Davis & Miles, 2000) or a preterm baby (Schappin et al., 2013; Siva et al., 2024), and meeting one's infant for the first time in the NICU after delivery (Nakazawa et al., 2006), were factors found to increase the mother's stress response. The final category, “Support Resources for mothers,” referred to human and institutional resources supporting the mothers. This included emotional (Suzuki et al., 2016) and financial support (Siva et al., 2024) from the family and others, as well as the NICU healthcare staff (D'Souza et al., 2009; Guillaume et al., 2013; Holditch‐Davis & Miles, 2000). It also encompassed community support, such as access to environments in which mothers can consult childcare professionals (Suzuki et al., 2016). A study conducted in India revealed that nursing support reduced maternal stress (D'Souza et al., 2009). Conversely, communication challenges with healthcare staff have been reported to increase maternal stress (Holditch‐Davis & Miles, 2000; Lee et al., 2005). In summary, support resources act as stressors that can have both positive and negative effects on mothers' mental health.

**TABLE 2:** Antecedents.

Category	Sub‐category	References no (corresponds to Table 1)
Admission of my infant to the NICU	My baby being in the NICU	3, 4, 10, 14, 15, 16, 22, 26
	Being separated from my baby	8, 21, 29
	Seeing my baby, a small, sickly, premature baby	13, 27
	Prolonged hospitalization	5, 12, 29
Physical environment of the NICU	Medical equipment and alarms	7, 10, 11, 13, 17
Barriers to parental role	Not being able to raise my baby the way I envisioned	10, 13, 29
	Inability to relieve pain	7, 29
	Inability to foster bonding	29
	Having to rely on information from the father	11
	Inability to touch my baby	11
	Having to suddenly change my life and plans	10
	Learning about new health care systems and Medical conditions	10
	Having to make critical decisions about my Baby's condition	10
Mother's characteristics	Maternal age	12, 26
	Low level of education	12, 13, 32
	Having psychological burdens	14, 32
	Economic constraint	29
Childbirth‐related experiences	Prenatal experience	13
	My baby, a premature baby	26, 29
	Face to face with my baby	31
Support resources for mothers	Relationships with Medical Professionals Support resources	6, 11, 13, 18, 29 21, 29

Abbreviation: NICU, neonatal intensive care unit.

### 3.3 Attributes

Three categories of attributes were identified: “Earnest feelings for my infant,” “Stuck feeling,” and “Vague anxiety about the future” (Table 3). The “Earnest feelings for my infant” category was defined as mothers' feelings of guilt for the preterm birth (Grieb et al., 2023; Holditch‐Davis & Miles, 2000; Lee et al., 2005), being overwhelmed by having their infant in the NICU (Grieb et al., 2023; Holditch‐Davis & Miles, 2000; Lee et al., 2005), and heartache (Holditch‐Davis & Miles, 2000). For example, Holditch‐Davis and Miles (2000) reported that some mothers expressed feelings of guilt for having given birth earlier than expected. In interviews with 13 mothers, Grieb et al. (2023) revealed that most felt guilty and overwhelmed. Furthermore, when their infants' condition deteriorated, mothers felt distant from their infants (Guillaume et al., 2013) and were traumatized by this experience (Holditch‐Davis & Miles, 2000). The present findings indicate a deeply connected relationship between the infant's condition and the mother's emotions. The “Stuck feeling” category encompassed feelings of frustration due to reliance on information from the father (Guillaume et al., 2013), feelings of not knowing what to do (Grieb et al., 2023; Hatters Friedman et al., 2013; Holditch‐Davis & Miles, 2000), and feelings of fear and anxiety (Hatters Friedman et al., 2013; Suzuki et al., 2016). Guillaume et al. (2013) found that a source of isolation and stress was frustration at not being able to see the baby for the first few days after birth and having to rely on the father for new information about the infant. Furthermore, mothers experienced difficulties in coping with unexpected situations (Hatters Friedman et al., 2013) and the appearance of a small and sick premature infant (Holditch‐Davis & Miles, 2000). The “Vague anxiety about the future” consisted of a sense of loss of control (Grieb et al., 2023) and uncertainty (Lee et al., 2005). This implies that the mothers felt out of control with respect to their vision of the future (Grieb et al., 2023), and had concerns about the infant's illness and its impact on the future (Lee et al., 2005).

**TABLE 3:** Attributes.

Category	Sub‐category	References no (corresponds to Table 1)
Earnest feelings for my infant	Overwhelming feeling	10, 13
	Feelings of guilt	10, 13, 18
	Feeling of being so far away from my baby	11
	Feeling heartache	13
	Traumatic	13
Stuck feelings	Frustration	11
	Feeling of not knowing what to do	10, 12, 13
	Anxiety and fear	10, 12, 13, 21
Vague anxiety about the future	Sense of loss of control	10
	Uncertainty	18

### 3.4 Consequences

Four categories of consequences were identified: “Bonding disorders,” “Persistent psychological symptoms,” “Partner relationship strain,” and “Discovering what only I can do” (Table 4). These four categories represent long‐term stress responses that occurred in mothers because of prolonged stress reactions. In the “Bonding disorders” category, some mothers reported difficulty developing emotional attachments with their infants after childbirth (Bonacquisti et al., 2020; Kim et al., 2020). Bonacquisti et al. (2020) reported a significant negative association between attachment and stress between mother and infant, suggesting that maternal stress negatively affects bonding with the infant. The “Persistent psychiatric symptoms” category revealed that mothers exhibited symptoms of PTSD (Aftyka et al., 2014; Aftyka et al., 2017; Hatters Friedman et al., 2013; Holditch‐Davis et al., 2003; Orovou et al., 2023; Schecter et al., 2020), postpartum depression (PPD; Levinson et al., 2022; Stotts et al., 2019), and post‐traumatic stress symptoms (PTSS; Shaw et al., 2009). One study reported the appearance of PTSD symptoms such as reexperiencing trauma, avoidance, negative thoughts and feelings, and arousal and reactivity in mothers of infants in the NICU (Orovou et al., 2023). Levinson et al. (2022) also found that higher stress scores in the NICU were associated with a higher rate of positive screening for PPD. Reportedly, psychological stress in the early stages of NICU admission is also a risk factor for the subsequent development of PTSS (Shaw et al., 2009).

**TABLE 4:** Consequences.

Category	Sub‐category	References no (corresponds to Table 1)
Bonding disorder	Postpartum bonding disorder	4, 17
Persistent psychological symptoms	PTSD	1, 2, 12, 14, 23, 27
	PPD	19, 30
	PTSS	28
	Presence of psychiatric symptoms at follow‐up	8, 15, 20, 27
Partner relationship strain	Strained relationship with partner	24
Discover what only I can do	Delivering breast milk	7, 25

Abbreviations: PPD, postpartum depression; PSS, Parental Stressor Scale; PTSD, post‐traumatic stress disorder; PTSS, post‐traumatic stress symptoms.

Not only did other psychiatric symptoms such as anxiety and depression persist up to 6 months postpartum at follow‐up (Lotterman et al., 2019), but mothers with high depressive and anxiety symptoms in the NICU remained in psychological distress 1 year after NICU discharge (Holditch‐Davis et al., 2015). In the “Partner relationship strain” category, as stress in the NICU increased, mothers became more likely to perceive tension in their relationship with the father (Reimer et al., 2023). In the “Discovering what I can do” category, it was revealed that even in situations in which the mother's role was limited, some mothers identified and acted on what only they could do—such as delivering breast milk (Rossman et al., 2013).

### 3.5 Surrogate term

“Psychological distress” and “psychological burden” were used to measure maternal stress. Unlike stress, these terms focus on purely psychological aspects.

### 3.6 Related concept

In the 32 references analyzed, “depression” (Levinson et al., 2022) was extracted as a concept related to maternal stress. Although this concept shares some similarities with maternal stress, it is independent and can be measured using specific scales. That is, this does not share the same set of attributes to which Rodgers refers, but it is a concept related to stress.

### 3.7 Definition of maternal stress in the NICU

Maternal stress in the NICU is caused by various stressors surrounding the mother and can be defined as a feeling of desperation for her infant, as well as feelings of inadequacy and vague anxiety about the future due to circumstances beyond her control. Moreover, maternal stress is cumulative, characterized not only by a direct response to the stressor but also by the effects on herself and her relationship with her infant and partner caused by the persistence of the stress response.

## 4 DISCUSSION

### 4.1 Characteristics of the concept

While systematic or scoping reviews integrate prior findings, they do not provide theoretical clarity. Concept analysis, in contrast, defines the essential attributes of a phenomenon. By applying concept analysis, this study not only synthesized the literature on maternal stress in the NICU but also clarified its definition, attributes, antecedents, and consequences. In the following, we discuss the characteristics of the concept revealed by this study from three perspectives.

#### 4.1.1 Antecedents: Stressors

The current results indicate that the NICU environment itself is a stressor for mothers. Unlike general hospital wards, NICUs are often open spaces where healthcare staff can respond to sudden changes in the affected infant from a distance. Prior studies have indicated that the absence of private space can be emotionally exhausting due to exposure to the problems of other families, even if they are not aware of them (Bry & Wigert, 2019). Furthermore, this study identified alarms as a significant source of stress for mothers (Guillaume et al., 2013). These findings underscore the need to create an environment where mothers can relax and engage with their infants.

In the “Barriers to parental roles” category, mothers experienced stress due to limitations on what they could do for their infants. Specifically, they could not engage in the caregiving they had envisioned, such as holding, breastfeeding, and bathing their infants (Holditch‐Davis & Miles, 2000), or have physical contact with their infants (Guillaume et al., 2013). Consistent with the findings of Miles et al. (1993), parents of infants admitted to the NICU commonly reported three major stressors: the infant's condition, changes in the parental role, and the environment. Among these, a meta‐analysis of parental stress in the NICU reported that “changes in the parental role” were the most significant stressor for parents (Caporali et al., 2020). This study also supported these findings by showing that the inability to fulfill the parental role serves as a major stressor for mothers.

According to Lazarus and Folkman (1984), stressors can sometimes have positive effects, such as fostering personal growth and motivating coping efforts. The present study demonstrated that “Support resources for mothers” can have both positive and negative impacts. While communication gaps with healthcare professionals (Siva et al., 2024) and the behaviors of these professionals (Holditch‐Davis & Miles, 2000) may cause anxiety in mothers, both physician and nurse visits to the bedside (Lee et al., 2005) and support from nurses (D'Souza et al., 2009) have been found to reduce stress levels. Previous research has reported that nursing support increases maternal empowerment (Maleki et al., 2022). Another study found a positive correlation between social support provided by nurses and mothers' satisfaction with their care (Eskandari et al., 2021), underscoring the need for health professionals to effectively address and regulate these stressors. Furthermore, factors identified beyond the “support resources for mothers” category may also buffer maternal stress. However, the 32 papers examined in this study did not describe any other factors, suggesting the need for further research to examine additional stress‐reducing factors in this field.

#### 4.1.2 Attributes and consequences: Stress responses

Postpartum, the maternal body experiences substantial hormonal changes. Levels of prolactin, which is responsible for milk production, and oxytocin, which is required for uterine contractions, increase. Meanwhile, levels of progesterone and estrogen, which are responsible for maintaining pregnancy, decrease (Walter et al., 2021). Evidence suggests that hormonal fluctuations during the postpartum period are associated with temporary mood changes, including maternity blues and PPD (Brummelte & Galea, 2016). Research has also indicated that postpartum women who have infants admitted to the NICU experience elevated levels of anxiety, depression, and stress compared to mothers of full‐term infants (Garg et al., 2023). Moreover, these forms of psychological distress are interconnected (Staver et al., 2021), indicating that maternal stress responses are not adequately explained by a single concept. The present study found that, in addition to anxiety and depression, a wide range of emotions was experienced by mothers, including guilt, uncertainty, and frustration. A salient feature of stress responses among NICU mothers also includes the presence of psychological distress rooted in their infant's condition, such as the emotional pain of being physically separated from their infant or the trauma associated with the fear of the infant's condition further deteriorating. For mothers, their babies are irreplaceable; even when a medical course is favorable, underlying stress related to the baby's condition is always present. Previous studies have reported that maternal stress symptoms can persist from admission to two to four months postpartum (Bonacquisti et al., 2020), indicating that NICU mothers are constantly experiencing some level of stress throughout the hospitalization period. Consequently, it is important for healthcare professionals who interact with mothers daily to go beyond superficial observations and recognize the unspoken, internal emotions that mothers may be experiencing. In this regard, the present study contributes to deepening healthcare professionals' understanding of the complex emotions experienced by mothers.

#### 4.1.3 Relationship with existing theoretical frameworks

The findings of the present study suggest that one stress theory, namely, that of Lazarus and Folkman (1984), can explain the stress experienced by mothers of infants in the NICU. First, the findings of the present study align with Lazarus and Folkman's (1984) theory that stressors and stress responses are dynamic processes. Here, stressors were categorized as antecedents, and stress reactions were categorized as attributes and consequences. Among these, the diverse stress responses arising from stressors differ in their mechanisms of occurrence, including stress responses directly generated by the stressor and those arising incidentally alongside it. In other words, maternal stress involves complex causality and is a dynamic process that can change over time.

Second, the current findings align with Lazarus and Folkman's (1984) theory that stressors and stress responses differ from person to person, even for the same stressor. According to Lazarus and Folkman (1984), stressors and stress responses are part of a process mediated by cognitive appraisal and coping. Therefore, even when exposed to the same stressor, the stress responses may differ, reflecting individual differences. In this study, the stress response of “discovering what only I can do” emerged as one of the identified outcomes. Some mothers who were unable to fulfill their role as parents experienced intense emotions and feelings of helplessness toward their infants, which resulted in a positive stress response to discovering what only they could do. This finding supports Lazarus and Folkman's (1984) stress theory and suggests that the same stressor may elicit a positive stress response in some individuals. These two findings indicate that maternal stress in the unique NICU environment can be explained within the framework of Lazarus and Folkman's (1984) stress theory.

The results of this study can be explained not only by stress theory but also by applying the Aguilera and Messick crisis model (Aguilera & Messick, 1982). According to this model, balance maintenance factors are important for maintaining psychological equilibrium in the face of stressful events, and their presence or absence can lead to crisis avoidance (Aguilera & Messick, as cited in Hazle, 1982). This study's findings can be explained by applying the crisis model; the results suggest that a psychological imbalance occurs when a mother experiences the stressful event of her infant's hospitalization, which leads to a crisis. Furthermore, based on this model, social support is a balance‐maintaining factor (Aguilera & Messick, 1982). That is, the support resources identified in this study correspond to social support in Aguilera and Messick's crisis model, which suggests that such support may lead to both positive and negative maternal stress reactions.

### 4.2 Limitations and implications for nursing

There are several limitations to this study. First, the procedure for selecting the literature needs to be considered. The literature was selected based on Rodgers' method of concept analysis. However, the initial screening was inadequate, resulting in the need to rescreen after a close reading of 45 articles (20%). The selection procedure would have been clearer and more reproducible if 223 papers had been included in the initial screening process. Second, the validity of the analysis must be considered. Although the analysis was conducted in collaboration with the second author to enhance credibility, the possibility that the interpretation was influenced by the researcher's subjectivity cannot be entirely ruled out. Third, the comprehensiveness of the literature search should be considered. The search terms, strategies, and time frame used in this study may not have fully captured all relevant research in this field. In particular, the number of articles in this area has increased since 2019, and because the literature search was conducted in September 2024, more recent studies may not have been included. Therefore, caution is required when applying these results to clinical nursing practices. As Rodgers conceptualizes phenomena as continually evolving (Rodgers & Knafl, 2000), the findings of this study should not be regarded as fixed. Future research incorporating broader and more up‐to‐date literature will allow for further refinement of the concept of maternal stress in the NICU.

However, the present study yielded a novel finding: among the various stressors, some have the capacity to help reduce maternal stress responses. These stress‐relieving factors include “relationships with healthcare professionals” and “support resources.” Nurses have a significant influence on the mothers of infants admitted to the NICU. Prior studies have demonstrated that various strategies employed by nurses, including individualized support programs and training initiatives, can effectively reduce the stress levels of mothers with infants in the NICU (Maleki et al., 2022). To enhance the effectiveness of interventions for mothers, nurses must improve their skills in assessing individual mothers' needs and providing personalized care. Although educational programs traditionally focused on maternal stress as an outcome, there is a growing need to develop educational programs that measure NICU nurses' assessment of individual mothers and their care skills as program outcomes.

Furthermore, family stress is one of the inhibitors of FCC (Abukari & Schmollgruber, 2024). Therefore, the development of an assessment tool for stressors experienced by mothers based on the findings of this study will allow for more accurate assessment and intervention to promote FCC in the NICU. The Parental Stressor Scale: Neonatal Intensive Care Unit (PSS: NICU), developed by Miles et al. (1993), is a well‐known tool for assessing maternal stressors. However, further research is needed to investigate the relationship between such scales and FCC. In today's society, understanding mothers' stress is essential to ensuring timely access to appropriate support when needed.

The findings of this study reinforce the relevance of existing programs, particularly Family Integrated Care (FICare), an evidence‐based and practical model grounded in the principles of FCC (Waddington et al., 2021). FICare is designed to support parents as primary caregivers by encouraging their active participation in the NICU, with healthcare providers acting as mentors and facilitators (Waddington et al., 2021). One of the maternal stress responses identified in this study—“Discovering what only I can do”—reflects a mother's attempt to find meaningful ways to fulfill her role despite the stress of the NICU environment. This response aligns directly with the core philosophy of FICare, which emphasizes empowering parents to participate in their infants' care. Moreover, FICare addresses a key stressor identified in this study—“Barriers to the parental role”—by facilitating opportunities for parents to engage in caregiving tasks and decision making. In summary, the primary contribution of this study is to provide evidence to support the promotion of FICare in NICUs.

## 5 CONCLUSIONS

Based on a concept analysis of maternal stress in the NICU, six antecedents, three attributes, and four consequences were identified. It was revealed that mothers in the NICU experience deep emotional attachment to their infants while also feeling helpless in situations beyond their control, and harbor vague anxieties about the future. Moreover, “Support resources for mothers” may positively influence mothers' psychological well‐being. Additionally, the consequence of “Discovering what only I can do” indicated that some mothers, despite experiencing stress, demonstrated a forward‐looking attitude. These findings imply that the NICU environment is not only a source of stress for mothers but can also function as a supportive space and an opportunity for them to reflect on their role as mothers.

## AUTHOR CONTRIBUTIONS

Mitsuki Nojima designed the study and collected data. Mitsuki Nojima analyzed data and wrote the paper. Hisayo Okayama critically reviewed the manuscript and supervised the entire process. All authors read and approved the final manuscript.

## CONFLICT OF INTEREST STATEMENT

The authors declare that there is no conflict of interest.
